# Breaking the mold with RNA—a “RNAissance” of life science

**DOI:** 10.1038/s41525-023-00387-4

**Published:** 2024-01-09

**Authors:** Charles H. Jones, John R. Androsavich, Nina So, Matthew P. Jenkins, Derek MacCormack, Andrew Prigodich, Verna Welch, Jane M. True, Mikael Dolsten

**Affiliations:** Pfizer, 66 Hudson Boulevard, New York, NY, 10018 USA

**Keywords:** Nucleic-acid therapeutics, RNA vaccines

## Abstract

In the past decade, RNA therapeutics have gone from being a promising concept to one of the most exciting frontiers in healthcare and pharmaceuticals. The field is now entering what many call a renaissance or “RNAissance” which is being fueled by advances in genetic engineering and delivery systems to take on more ambitious development efforts. However, this renaissance is occurring at an unprecedented pace, which will require a different way of thinking if the field is to live up to its full potential. Recognizing this need, this article will provide a forward-looking perspective on the field of RNA medical products and the potential long-term innovations and policy shifts enabled by this revolutionary and game-changing technological platform.

## Introduction

Our perception of the RNA molecule’s role in biology has come a long way since we initially believed it to be a passive go-between for DNA and protein. Through years of research and countless discoveries, we now recognize RNA as a vital, dynamic, and active component in various biological processes. This understanding, along with concomitant discoveries over the last couple of decades, positioned RNA-based therapeutics as the next promising “drug of tomorrow.”

RNA’s potential is largely due to its difference from conventional drug strategies. Unlike conventional drugs, which typically bind to active sites on proteins to alter their function for therapeutic response, RNA-based therapeutics can target any gene in the genome, even genes that do not encode for proteins but are still implicated in disease (e.g., non-coding RNA)^1^. This is notable because proteins constitute only 1.5% of the human genome, and only ~10–14% of those proteins have accessible drug-binding sites^2^. Furthermore, the nucleotide sequence in RNA-based therapies is responsible for targeting, with sequence editing requiring minimal or no modifications to the production process^1^. The production of RNA-based therapies is thus flexible and fast, with the editing of sequences often as straightforward as inputting the new sequence into computer software connected to a synthesizer. These attributes of RNA land RNA-based platforms in the category of “programmable drugs” alongside gene-modified cell therapies and other DNA-based gene therapies. These drugs currently only make up ~8% of the 340 approved biologics, which are, in-turn, only a small fraction of conventional drugs^3^. These features, along with research in harnessing RNA for therapies, set the stage for the success of mRNA vaccines during the COVID-19 pandemic.

### Historical overview

**Fig. 1:**
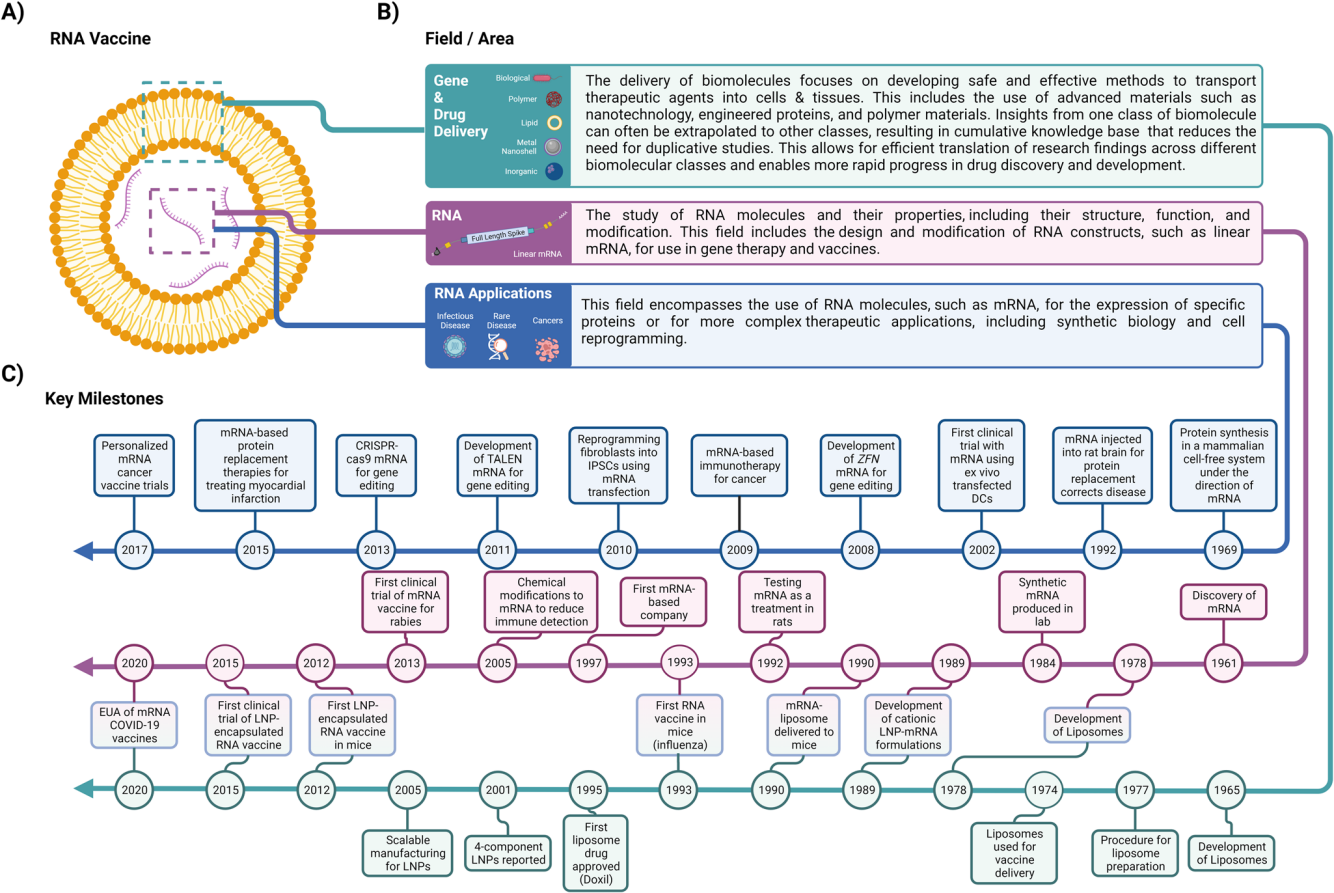
mRNA vaccine development: structure, research fields and areas, and key milestones. **A** mRNA vaccine. Schematic representation of an mRNA vaccine illustrating the lipid bilayer encapsulating the single-stranded mRNA. **B** Research fields and areas. An overview of the key research fields associated with mRNA vaccines, comprising Gene & Drug Delivery, RNA biology, and RNA applications **C** Key milestones^13^. Major advancements in three fields/areas, gene and drug delivery, RNA, and RNA applications, led to the approval of the first mRNA vaccines. RNA application milestones highlight RNA’s expansion into new therapeutic areas, including vaccines to target tumors; delivery of novel protein therapeutics and gene replacement therapies; cell lineage reprogramming; and gene-editing technologies. CRISPR clustered regularly interspaced short palindromic repeats, EUA emergency use authorization, IPSCs induced pluripotent stem cells, LNP lipid nanoparticle, mRNA messenger RNA, NP nanoparticle, sgRNA single guide RNA, TALEN transcription activator-like effector nucleases. “Created with BioRender.com”.

Despite becoming a household name in 2020, mRNA vaccine development did not happen overnight^1^. The discovery of messenger RNA (mRNA) occurred in the 1960s^4^. However, it was not until the 1990s that mRNA was explored as a possible therapeutic^1,5^. Pharmaceutical industry leaders, before the pandemic, saw the potential of mRNA technology and invested in its development. For instance, in 2018 Pfizer partnered with BioNTech to collaborate and develop an mRNA platform for influenza vaccines (Supplementary Information, Box 1)^6^. Despite initial concerns about mRNA’s instability and high production costs, scientific breakthroughs in mRNA and carrier lipid nanoparticles (LNPs) have helped overcome the previous limitations and enabled substantive progress to be made in the delivery, manufacturing, safety, and synthetic modification of mRNA (Fig. 1A). The culmination of several decades of research in the field of gene and drug delivery contributed in the success of RNA-based platforms^7^. This work led to a deeper understanding of RNA structure interactions within cellular systems, which in turn enabled scientists to optimize endosomal escape, translation, and degradation processes, while minimizing immune system recognition and inflammatory responses^8–12^. As a result of these advancements, mRNA technology was well-positioned to showcase its capabilities in the fight against COVID-19.

### COVID-19 pandemic as a real-world proof of concept

While the research supporting mRNA vaccines has been underway for decades^13^, the COVID-19 pandemic presented a real-world situation that allowed for the rapid evaluation of mRNA vaccines within the global population. Key partnerships accelerated the application of mRNA technology in developing and deploying vaccines at scale. In less than a year after clinical trials had started, two COVID-19 mRNA vaccines received emergency use authorization from the Food and Drug Administration (FDA). Several environmental factors also enabled the rapid rollout of mRNA vaccines, including increased funding, improved global coordination resulting from previous outbreaks, and other emergency approvals^14,15^.

Moving forward, the therapeutic potential of mRNA will expand beyond vaccines, driven by its versatility. Given the recent validation of mRNA technology and current advancements in RNA technology, changes across the entire RNA medical product (RMP) landscape are anticipated soon, including the reexamination of regulatory pathways and changes in commercial models for pharmaceutical companies. To better prepare for this future, we will provide insights into the future of RMPs, emphasizing the advancements in RNA biotechnology, the expansion of therapeutic RNA molecules, and the increased investments and intellectual properties. In addition, we will explore how the growth of RMPs might influence the pharmaceutical ecosystem.

### RNA primer

#### Overview of RNA, RMPs, and their mechanisms of action

RNA is a biomolecular class with a broad array of biological functions and represents a rapidly growing category of drugs with the potential to greatly influence prophylactic and therapeutic approaches in medicine. In addition to coding for proteins, RNA plays a critical role in the regulation of gene expression, post-translational modifications, and splicing^16^. This versatility makes RNA an attractive molecule for drug development and has resulted in a range of effective RMPs. For this perspective, we will outline the six main categories of RNA-based therapeutics and vaccines: antisense oligonucleotides (ASOs), small interfering RNAs (siRNAs), microRNAs (miRNAs), aptamers, mRNAs, and CRISPR, which uses guide RNAs (gRNAs) for targeting (Table 1)^2^.

**Table 1:** RNA-based therapeutic and vaccine modalities.

Type	MoA(s)	Advanced program	Clinical pipeline
**ASOs**	• Single-stranded oligonucleotides^1,2,136^ • Promote mRNA degradation and target silencing through cleavage of a target RNA sequence using RNase H activity^1,2,136^ • Use a steric hindrance–based mechanism to alter other functions (e.g., alter splicing of pre-mRNAs to selectively include or exclude certain exons)^1,2,136^	**Marketed** • Nine therapeutics have received approval though two have since been withdrawn^101^ • First approval in 1998, though this was later withdrawn^101^ Examples **Inotersen (SC)**: Induces degradation of transthyretin mRNA in the liver to reduce circulating protein levels for the treatment of hereditary transthyretin amyloidosis^1,137^ **Golodirsen (IV)** and **eteplirsen (IV)**: Restore mRNA reading frame of dystrophin pre-mRNA to facilitate functional protein production for the treatment of Duchenne muscular dystrophy^138,139^ **Nusinersen (IT)**: Binds to *SMN2* pre-mRNA to promote exon 7 inclusion and increase SMN protein production for treatment of spinal muscular atrophy^140^	• At least 50 therapeutics in Phase II or Phase III clinical trials^101^ • Investigational therapeutics target numerous indications, including metabolic disorders, cancer, and respiratory conditions^101^
**siRNAs**	• Double-stranded molecules typically 19 to 21 nucleotides long^136^ • Function as sequence-specific guides to suppress mRNA expression using the endogenous RNAi pathway^1,136,38^	**Marketed** • Four marketed therapeutics and one in preregistration^101^ • First approval in 2018^101^ Examples **Patisiran (IV)**: Reduces transthyretin protein production to reduce amyloid deposits for treatment of hereditary transthyretin-mediated amyloidosis^1,56^ **Givosiran (SC)**: Lowers production of ALA synthase 1 to normalize levels of heme biosynthesis for treatment of acute hepatic porphyria^1,54^ **Inclisiran (SC)**: Decreases translation of liver PCSK9 to reduce LDL cholesterol levels for treatment of primary hypercholesterolemia or mixed dyslipidemia^1,141^	• At least 25 therapeutics in Phase II or Phase III clinical trials^101^ • Investigational therapeutics target numerous indications, including metabolic disorders, infectious diseases, and cancer^101^
**miRNAs**	• Endogenous short non-coding RNAs^2^ • Share the same RNAi machinery as siRNA but can regulate expression of multiple target genes^2^ • Can be inhibited using ASOs or supplemented for gain-of-function effect^2^	**Marketed** No approved therapeutics to date	• At least 10 therapeutics in Phase I, Phase II, or Phase III clinical trials^142^ • Investigational therapeutics target different indications, including wound healing, heart failure, T-cell lymphoma, liver cancer, hepatitis, and glioblastoma^2,143^
**Aptamers**	• Single-stranded oligomers consisting of 20 to 100 bases^1,2^ • Engineered to bind protein targets based on their tertiary structure to modulate function^1,2^ • Analogous to the RNA version of monoclonal antibodies^2^	**Marketed** • Two approved therapeutics to date but production has since been discontinued for one of them^142^ • First approval in 2004^57^ Example **Defibrotide (IV):** Activates adenosine A1/A2 receptor for treatment of veno-occlusive disease in the liver^142^	• At least two therapeutics currently in Phase I or Phase II clinical trials • Investigational therapeutics are currently in development for various indications, including diabetic nephropathy, pancreatic cancer, and glioblastoma/glioma^2^
**mRNAs**	• Can be used to express any protein, wild-type, or imagined^1,53,36^ • Rescue or supplement function • Antigen to stimulate an immune response • Effector protein to edit the genome or epigenome • Transcription factor to alter cell state • Chimeric antigen to program the immune system against disease	**Marketed** • Two approved vaccines^142^ • Emergency Use Authorization in 2020 Examples **Tozinameran (IM)** and **elasomeran (IM)**: Elicit translation of a modified spike protein to induce a SARS-CoV-2–specific immune response for the treatment of COVID-19^2^	• >20 therapeutics and vaccines currently in Phase I, II, or III clinical trials^144^ • Investigational therapeutics target numerous indications, including infectious diseases and cancer^144^
**gRNA/ CRISPR**	• Used for gene editing via RNA-guided DNA cleavage then repair at a target DNA site, allowing for specific corrections, alterations, additions, or deletions to the genome^145^ • Engineering gRNA can improve the system’s specificity, stability, and safety and expand their applications^145^	**Marketed** No approved therapeutics to date	• >25 therapeutics currently in Phase I, II, or III clinical trials^21^ • Investigational therapeutics target numerous indications, including cancer, sickle cell disease, diabetes, HIV, and hereditary disorders^21^

ALA delta-aminolevulinic acid, *ASO* antisense oligonucleotide, *CoV* coronavirus, *CRISPR* clustered regularly interspaced short palindromic repeats, gRNA guide RNA, *HIV* human immunodeficiency virus, *IM* intramuscular, *IT* intrathecal *IV* intravenous, *LDL* low-density lipoprotein, *miRNA* microRNA, *MoA* mechanism of action, *mRNA* messenger RNA, *PCSK9* proprotein convertase subtilisin/kexin type 9, *RNAi* RNA interference, *siRNA* small interfering RNA, *SARS* severe acute respiratory syndrome, *SC* subcutaneous SMN survival motor neuron, *VEGF* vascular endothelial growth factor.

The general mechanisms of action (MoA) of the major RNA modalities are well established at the molecular level and have been skillfully reviewed in detail by others^17–22^. Summaries for each MoA are provided in Table 1 for reference. An RMP’s precise MoA is specific to the target of interest, indication, and relevant site of action, which can vary widely given the broad and diverse applications of RNA. The approved COVID-19 mRNA vaccines, as one example, encode the SARS-CoV-2 spike surface glycoprotein locked in a prefusion conformation that mimics the viral attachment protein that resides on the outside surface of viral particles prior to infection^23,24^. Upon intramuscular (IM) administration, the spike-encoded, nucleoside-modified mRNA delivered inside of an LNP distributes to nearby cells proximal to the injection site and in adjacent draining lymph nodes^25,26^. Upon entering the cytoplasm of these cells, the mRNA is translated by ribosomes, resulting in protein that is subsequently processed through antigen presentation pathways^27^. The presentation of foreign antigen by the major histocompatibility complex (MHC) class I or II on the cell surface, along with a pro-immunogenic cytokine effect induced by the mRNA and LNP themselves, triggers host immunity and protection through humoral and cellular responses^17,25,28,29^. While the contributions of antigen expression by muscle cells directly transfected with mRNA/LNP remain unclear^26^, professional antigen-presenting cells (APCs), such as dendritic cells, more clearly play a central role in the immune response elicited by mRNA vaccines. APCs which have taken up mRNA/LNP directly or antigen indirectly, traffic to lymph nodes where they prime CD4 and CD8 T lymphocytes and initiate germinal center reaction through T follicular helper cells, resulting in the generation of memory B cells and antibody-producing plasma cells^25,28,30^.

The path to effective RNA-based therapeutics has been an iterative process, with each advancement for a particular approach informing design improvements for the other approaches^31^. Early research aimed at improving the stability of ASO-based therapeutics revealed chemical modifications that could enhance delivery, which were then applied to aptamer- and siRNA-based therapeutics^31–33^. The development of LNPs to deliver siRNA therapeutics provided a feasible mechanism for the delivery of large mRNAs, leading to their use in mRNA-based vaccines against SARS-CoV-2. Currently, advancements in LNP delivery systems and chemical modifications of single-stranded RNA are being combined to ensure efficient delivery of mRNA with gRNAs for CRISPR-Cas9 gene editing^34,35^. This interconnected nature of RNA innovation has resulted in a vast number of new RNA drug candidates currently under development. Further advancements, particularly in design and delivery systems, will be crucial for the continued proliferation of RNA technology into the future^2,36^.

#### Advancements in RNA sequence design and structure

RNA is composed of a selection of four nucleobases attached to a five-carbon sugar linked together by a phosphate backbone in an elongated chain of varying lengths. Over time, this prototype has been altered with a series of chemical modifications to the sugar, base, and backbone to optimize pharmacokinetics, biodistribution, safety, and potency of RMPs. While it is beyond the scope of this work to provide a comprehensive overview of RNA design, it is suffice to say that well-established design archetypes exist for all modalities^36,37^, such as the 5-10-5 gapmer for ASOs^38^, the alternating 2’-deoxy-2’-fluoro and 2’-O-methyl motifs for siRNA^39^, and the codon-optimized mRNA incorporating N1-methylpseudouridine^40^. However, the state of the art continues to evolve into more sophisticated and diversified designs, and the RNA design space is extraordinarily large, with countless possible combinations in sequence and chemical space.

Optimal oligonucleotide (siRNA, ASO, gRNA) sequences are selected by “walking” along gene targets using cell-based assays. By screening a library of each possible oligonucleotide sequence complementary to the target, those with the highest knockdown or editing activity are selected. This is often aided by computational algorithms that remove candidate oligonucleotides, a priori, that are predicted to perform poorly^38^. Once top hits are identified, their chemistries are further optimized using structure-activity relationship (SAR) to produce lead candidates. However, it should be noted that certain combinations of chemical modifications and oligonucleotide sequences must be carefully optimized to avoid hybridization-dependent and independent toxicity^41,42^.

In contrast to oligonucleotides, mRNA designs are more flexible, and a variety of sequence design approaches are being developed to enhance mRNA attributes. Table 2 summarizes major approaches, including codon optimization, nucleic acid modifications, and polyadenylation. For example, although the open reading frame (ORF) of mRNA is fixed to encode a specific target protein sequence, codon optimization can be used to select codons that facilitate the highest potential protein output^43^. Additionally, nucleic acid modifications, such as the uridine substitution for N1-methylpseudouridine in COVID-19 mRNA vaccines^44^, can improve stability and protein expression while decreasing immunogenicity. Over 20 unique chemical modifications found in endogenous eukaryotic mRNAs could be used in the future to further improve modified RNA (modRNA) therapeutics^45^. Importantly, limited chemical or sequence changes made to an mRNA are less likely to have severe safety risks, especially if the changes do not alter the size or secondary structure of the new mRNA or its interaction with the lipid nanoparticle (LNP)^46^. This makes mRNA a desirable modular platform from a regulatory standpoint, which will be discussed in a later section.

**Table 2:** Common linear mRNA sequence modifications^53^.

Region	Modification	Impact on function
5’ cap	Methylation	Reduce immunogenicity and enhance translation
5’ UTR	Incorporation of IRES viral sequences	Initiate translation at lower initiation factor expression levels to improve efficacy
ORF	Codon optimization	Several modifications (such as using more frequent codons, using codon pairs that work better together, using the same proportion of each codon found in highly expressed proteins, or reducing UU and UA dinucleotides) can increase rate and efficiency of translation but may impact function or alter conformation
3’ UTR	Incorporation of specific α- or β-globin mRNA sequences	Addition of α-globin sequence improves stability while β-globin sequence extends duration of protein expression
Poly(A) tail	Increased length	A long poly(A) tail can increase stability and efficiency of protein translation but should be optimized for each target cell type
	Incorporation of adenosine analogs	Protect mRNA from 3’-exonuclease activity
Any	Modification of nucleosides	Optimize protein expression through uridine depletion Lower innate immune responses induced by Toll-like receptor by incorporating 5-methylcytidine, N^6^-methyladenosine, or 5-methyluridine

*IRES* internal ribosome entry site, *mRNA* messenger RNA, *ORF* open reading frame, *UTR* untranslated regions.

Despite modifications and sequence design improvements, mRNA is still generally a labile molecule that may require repeat administration to achieve the required level or duration of expression^47^. New types of RNA, including self-amplifying mRNA (saRNA) and circular RNA (circRNA), could address stability limitations^48^.

Encoded viral-derived RNA replication machinery carried by saRNA can copy itself once delivered inside the cell, allowing for higher and longer-lasting expression of antigens relative to the amount of mRNA delivered to the cell^2,47^. In vaccine development, saRNA may potentially reduce the vaccine dose required for generating protective immunogenicity^2^, which is beneficial during pandemics when drug product is scarce. The technology behind circRNA uses condensing agents, ligases, or self-splicing introns to circularize mRNA into a continuous loop by forming a covalent linkage between 5’ and 3’ ends^49^, resulting in a more stable molecule with a longer lifespan compared to mRNAs^50^. Due to their closed-loop structure, circRNAs are inaccessible to exonucleases and resistant to mRNA turnover via CCR4-NOT1- or DCP-dependent decay^49,51^. In addition, internal ribosome entry sites (IRES) used to promote protein translation might provide additional control for tuning therapeutic activity in a cell-dependent manner^52^. Different RNA formats offer opportunities for more complex future applications, such as monoclonal antibody expression, multivalent vaccines, and multifactor complexes.

#### Progress and challenges in delivery and formulation of RNA therapeutics

Effective delivery systems are important for the success of RMPs. Due to RNA’s high molecular weight, hydrophilic nature, and negative charge, it poorly diffuses across cellular membranes on its own^2,53,36^. While single-stranded ASOs can penetrate certain cells through clathrin- or caveolin-dependent endocytic pathways or nonconventional endocytic pathways^37^, double-stranded siRNA requires specialized formulations such as LNPs or conjugation to ligands like N-acetylgalactosamine (GalNAc) for targeted cellular uptake^54,55^. For example, conjugating GalNAc to siRNA resulted in the effective delivery of the FDA-approved givosiran to the liver^56,52^. ASOs also benefit from similar conjugation strategies that enhance potency or redirect biodistribution.

For mRNA therapeutics and vaccines, encapsulation in carrier systems is often required for cell uptake and protection from degradation by ribonucleases^2,57,53^. These systems use biomaterials^36^, biological methods^58^, and physical methods^59^ for RNA delivery.

Biomaterial-based delivery systems include polymer-, lipid-, and peptide-mediated approaches. Polymer-based delivery systems use cationic polymers (poly-L-lysine, polyethylenimine, etc.) to form polyplexes with negatively charged mRNA, creating a stable delivery system that is easy to prepare, purify, and modify; it also protects mRNA from ribonucleases^36^. However, a key challenge with polymers is poor biodegradability, which poses toxicity from accumulation in the cells^60^. LNPs are currently used for larger RNA molecules^36^, as they provide enhanced RNA delivery and minimize degradation^53^. However, further research is required to ensure efficient delivery of liposomes while minimizing degradation in the liver or kidney for therapeutic applications^1,53^.

Biological methods include viruses, viral-like particles (VLPs), and extracellular vesicles (ECVs). Adeno-associated virus (AAV) can be used to deliver RNA payloads such as short hairpin RNA (shRNA) and gene-editing cargos^61,62^. These are attractive systems for gene therapy as they naturally infect primates and are nonpathogenic. However, their clinical use has many obstacles including potential genomic integration, immunogenicity, packaging size limits, preexisting immunity, limited upscaling, and expensive manufacturing methods^58,61,62^. VLPs resemble native viruses but lack pathogenicity and are used for delivery because of their biocompatibility, biodegradability, and targeting ability^53,36^. Use of ECVs, such as exosomes, for RNA delivery also elicits enhanced cellular uptake, reduced toxicity, and shows no liver accumulation of the RMP^53^. Although manufacturing challenges currently limit commercial scale applications for both VLPs and ECVs^63^, valuable learnings can still be applied to the design of standard formulations.

Compared to the above methods, physical methods like electroporation are particularly effective for introducing tumor-associated antigens or chimeric antigen receptors (CARs) that can reprogram cell-based therapeutics ex vivo, such as CAR-T cell therapies, without risk of genomic integration and with decreased immunogenicity^59,64^. However, some limitations such as cytotoxicity caused by disruption of cell membrane integrity, cost, and infrastructure, could make physical methods a less practical option for clinical applications.

Another focus in mRNA delivery is enabling selective targeting of certain cell types and tissues beyond the liver. Current strategies include passive and active targeting. Passive targeting involves modifying the size and shape of the LNP. In contrast, active targeting involves incorporating specific components onto the LNP surface that can selectively drive delivery to target cells, such as macrophages, T cells, and B cells^65–67^. This can include adding a selective organ targeting (SORT) molecule to allow for extrahepatic delivery following IV administration^68^. SORT molecules alter the LNP charge, apparent pKa, and serum protein interactions, enabling selective targeting of clinically relevant cell types beyond the liver, including epithelial and endothelial cells, T cells, B cells, and hepatocytes^69^. Specific cell-targeting antibodies or other moieties (such as polyglutamic acid functionalized with di-mannose for tumor-associated macrophages) will have far-reaching implications by reprogramming T cells without the need for ex vivo manipulations^66,70^. These LNP surface modifications can also be coupled with the addition of miRNA target sites to the mRNA to limit translation in off-target cell types^71^.

As more RNA-based therapeutics are developed, expanding administration routes will be important. While intravenous delivery is used to broadly target multiple organs, administration of therapeutics intranasally, intrathecally, or subcutaneously may increase therapeutic effectiveness for certain diseases^58^. Intranasal administration is a promising area of research, allowing access to the central nervous system without having to cross the blood–brain barrier^72,73^. This can also be accomplished with intrathecal administration, as seen with the approved ASO nusinersen^31^. Administration by inhalation is another area of intense research in order to target lung diseases without systemic exposure^74^. These alternate methods for administration could be transformative by easing patient access and allowing home administration of RNA-based therapeutics.

#### Expansion of surrounding fields

Gene editing has seen notable progress alongside mRNA research, such as mRNA delivery of editing technologies (TALENs [transcription activator-like effector nucleases], CRISPR/Cas9), which allows for temporary expression and minimal risk of genome insertion compared to protein or plasmid DNA approaches^36^. Advancements in gene editing and mRNA are starting to converge to push one another forward even further. The distinction between gene-editing therapeutics and mRNA therapeutics and vaccines is rapidly blurring as mRNA/LNP has become the preferred modality for delivering in vivo CRISPR and other gene-editing cargos^75^. This harmonization of pathways for gene editing using mRNA/LNP requires expertize and investments in both disciplines. In the future, mRNA platforms will likely advance as a tool for more complex treatments, where the therapeutic outcome is not limited to expressing a missing protein but instead initiates a cascade of biological activities with the translated protein.

RNA nanotechnology is also an expanding area of research that is poised to revolutionize various fields, including synthetic biology, diagnostics, and therapeutics. These RNA nanoparticles open up new possibilities for treating diseases that are currently difficult to manage, such as cancer and genetic disorders. For instance, researchers have used RNA nanoparticles to deliver therapeutic molecules to tumor cells, effectively killing them without harming healthy tissue^76–78^.

Moreover, RNA nanoparticles can be engineered to have a variety of shapes and sizes, allowing for precise control over their properties. This versatility makes them ideal for use in drug delivery, where they can be designed to carry a wide range of therapeutic agents and deliver them to specific locations in the body^79,80^.

In addition to their potential in medicine, RNA nanoparticles also have applications in biotechnology. For example, they can be used to control gene expression, providing a powerful tool for studying biological processes and developing new treatments for diseases^81^. Furthermore, RNA nanoparticles can be used to construct complex nanostructures, such as nanomachines, which could have a wide range of applications in fields like nanomedicine and materials science^82^.

However, the field of RNA nanotechnology also faces several challenges. One of the main obstacles is the difficulty of large-scale production of RNA nanoparticles. While several methods have been developed for producing these particles, they are often time-consuming and expensive^83^. Moreover, there are also concerns about the stability and safety of RNA nanoparticles, which need to be addressed before they can be widely used in clinical applications^83–87^.

### Looking toward the future

The RMP landscape is quickly evolving on the other side of the COVID-19 pandemic. Before, the commercial success of RNA drugs was mostly limited to a modest number of transformative specialty products developed for treating rare diseases^88^. Now, the success of mRNA vaccines has provided the wider platform validation necessary to fuel growth in the industry to develop products in all categories for both small and large demographics^89^. Resources are also being funneled into targeting other therapeutic areas beyond vaccines, such as neurological and metabolic diseases, continuing to expand the RMP landscape^2,89^.

To better assess the current growth and activity of RNA products in the market, we highlight findings from a meta-analysis using biopharma intelligence databases to assess trends across commercial sales, intellectual property (IP) filings, investments, and the number and type of preclinical and clinical RNA programs under investigation over the last 5–12 years.

**Fig. 2:**
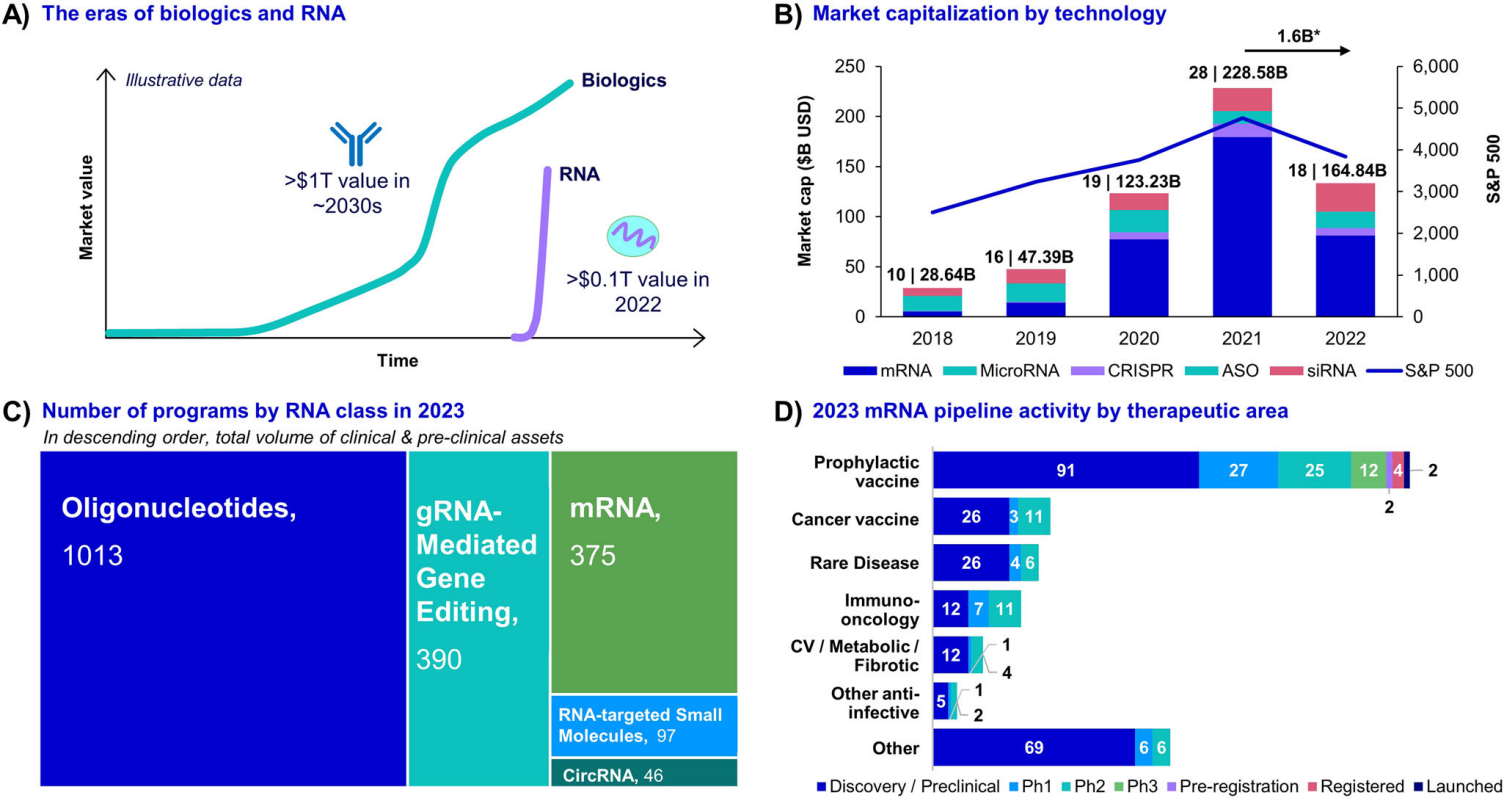
Current RMP market. Meta-analysis of the current RMP space lends visibility into the level of activity in the field as well as its recent trends. **A** The eras of biologics and RNA. Illustrative data shows the rapid market capitalization growth rate RMPs contrasted with the comparatively slower market capitalization growth rate of biologics. **B** Market capitalization by technology. The total market capitalization of companies with RNA technology assets (excluding diversified companies, such as Merck, Pfizer, and Sanofi) from 2018 to 2022 is broken down by RNA technology and is compared to the S&P 500. Numbers above each bar represent the number of companies included in the analysis and the total market capitalization for that year. The market capitalization for publicly traded companies was estimated by multiplying historical closing share prices on December 31^st^ for each year by the outstanding shares. This data was obtained from Yahoo Finance, accessed May 17, 2023. The data for the S&P 500 was obtained from Yahoo Finance and accessed May 17, 2023. Standard statistical methods were used in the analysis of the data. *Some 43 private companies working with mRNA raised ~ $1.6B in funding from 2021 to 2022. **C** Number of programs by RNA class in 2023. The total volume of preclinical and active clinical programs in January of 2023 is represented in descending order. Data was obtained from Beacon Intelligence, RNA dataset, accessed April 1st, 2023. Data View used in the RNA dataset was ‘Drug Data.’ **D** 2023 mRNA pipeline by therapeutic area. The number of mRNA programs in 2023; all phases of development are broken down by therapeutic area. Data was obtained from Beacon Intelligence, RNA dataset, accessed April 1st, 2023. Data View used in the RNA dataset was ‘Drug Data.’ *ASO* antisense oligonucleotide, Cap capitalization, CircRNA circular RNA, CV cardiovascular, gRNA guide RNA, Ph phase, siRNA small interfering RNA.

The growth in market capitalization of RMPs in recent years is remarkable, especially when compared to that of biologics. As of 2022, the total RMP market capitalization has surpassed $0.1 T, making it one of the most rapidly growing segments of modern medicine (Fig. 2A). Nusinersen, an ASO treatment for spinal muscular atrophy, a rare neuromuscular disease affecting children, has emerged as the top seller, with peak sales of $2.1B in 2019, three years after receiving approval in 2016. In the larger adult segment, inclisiran is the first siRNA approved for treating dyslipidemia in patients at high risk for cardiovascular disease. While the drug has had a slow initial uptake in the region, it is expected to reach sales of $2.7B by 2028.

In contrast to the slow and steady growth of RNA therapeutics, COVID-19 vaccine sales skyrocketed, reaching over $62.6B in just one year. While the pandemic has clearly created a unique period for elevated sales, billions more in revenues from COVID-19 vaccines are predicted into the foreseeable future^90^. Furthermore, the now established and favorable development and regulatory path for RNA vaccines will likely lead to additional prophylactic products for protecting large demographics from infectious diseases (Supplementary Fig. 1). Despite growth in both categories, RNA vaccines are predicted to maintain their lead over RNA therapeutics in terms of risk-adjusted revenue for at least the next 10 years^90^.

Trending with commercial sales, RNA-related IP filing activity has also been on the rise since 2015, reflecting increased precommercial-stage growth and innovation (Supplementary Fig. 2), although the filings remained flat from 2021 to 2022. High-volume patent filings are in part fueled by a highly competitive landscape, which promotes a mindset of “file early and file often.” With several high-profile patent disputes in courts^91^, the pressure to innovate and build strong IP portfolios has never been more important. It has also never been more complicated as RMPs are often multicomponent drug products that require several technologies and innovations to be combined for safe, efficacious delivery and activity. As a result, the number of patent applications filed on RNA technologies, methods of use, and compositions is likely to continue to increase.

The growth rate in the market capitalization of RNA-based pharmaceutical companies (excluding diversified companies, such as Merck, Pfizer, and Sanofi) starting in 2020 and topping $228B USD in 2021 (Fig. 2B) comes as no surprise following the highly visible success of RNA vaccines. This is remarkable since only 10 years ago, market capitalization for this class of drugs was under $3B USD. The potential for growth has attracted new and existing players who have seized the opportunity to raise capital, with 43 private companies working in mRNA raising $1.6B over the last 12 months, according to Pitchbook. Significantly, RNA technology is no longer limited to smaller private companies. All major pharmaceutical companies now have capabilities in RNA technology through new partnerships mergers, and acquisitions. In a short period of time, the RMP market went from limited commercial viability to demonstrating rapid expansion, investing in a broader array of therapeutics, and being populated with leading pharmaceutical companies with expertise in drug development.

Now that public and private markets have cooled, the biotech sector will generally need to adjust, focusing on leaner growth to prolong raised cash until the demonstration of clinical proof of concept for lead assets. Despite this, RNA-related technologies seem to be displaying resilience to the downturn. In 2022, Prime Medicine, a CRISPR/gRNA gene-editing company, raised $175 M in the bear market^92^. This was the largest initial public offering (IPO) for a preclinical company in 2022, suggesting that investors remain positive about the prospects of early-stage RNA technologies, especially gene editing. Momentum built over the last 12 months will also help position RNA going forward. In addition to an 87% growth in preclinical programs, there were also 56% and 53% increases in approvals and clinical programs, respectively. This signals RNA candidates can successfully meet milestones to reach the clinic and eventually commercialization (Supplementary Fig. 3).

The RNA landscape is also trending toward a more balanced distribution of product candidates, which will help diversify the collective portfolio, therefore helping to hedge against the inevitable drawback and any perceived read-through as well as driving sustainability and growth. While oligonucleotides like ASOs and siRNAs still make up most current programs, there has been an increase in mRNA, CRISPR/gRNA, and other technologies, both in absolute numbers and as a share of the field (Fig. 2C, Supplementary Fig. 4). The mRNA pipeline, in particular, has focused on areas with proven paths to revenue that are similar to that of prophylactic vaccines, such as cancer vaccines and immune-oncology therapeutics. It has also focused on areas with established technological feasibility, such as therapeutics that can deliver a systemic response within the body, and has neglected more technologically challenging areas, such as developing therapeutics that deliver targeted response to organs (Fig. 2D).

RMPs offer new prophylactic and therapeutic approaches that are flexible, fast, and comparatively inexpensive to manufacture. However, the cost of goods (COGs) associated with RNA therapeutics is a complex issue that goes beyond production costs. While it is true that advancements in RNA technology and delivery systems have reduced some production costs and accelerated development, there are numerous factors that contribute to the overall COGs. For instance, the fill-and-finish costs for the COVID-19 vaccines have been estimated to range from US$ 0.15 to US$ 0.30 a dose, based on 100 million doses a year^93^. Accounting for the cost of building and equipping a vaccine plant, staffing the plant, and producing the vaccine substance, this cost rises to between $0.54 and $0.98 per dose^93^.

The precision required for timing and location of therapeutic effects, the need to overcome supply chain and refrigeration challenges, the difficulties associated with disease-specific issues, and the capacity to formulate intricate biomolecules all add to the costs. Furthermore, each unique RNA therapeutic product requires rigorous monitoring of parameters such as stability, identity, sterility, encapsulated mRNA quantification, and size distribution of particles^94,95^. The COGs also include the expenses related to increased manufacturing capacity, thanks to privately owned supply chains and contract development and manufacturing organizations (CDMOs). Despite the relative ease and flexibility of RNA manufacturing, the investment needed to establish and maintain these facilities is substantial^96^. Lastly, the development of platform advancement strategies, such as limited delivery vectors, can accelerate drug development but also add to the costs. While these strategies can leverage existing data packages and focus on the assessment of the encoded target, clear guidelines from regulators are yet to be established^97^.

### An impending revolution?

The substantial growth and activity of the RMP industry foreshadows changes to the healthcare system. To stay ahead of the curve, stakeholders should ask thoughtful questions on how the combination of rapid pace of development and diversification of technologies will impact processes downstream. For example, platform-based drugs hold the promise of faster and more frequent development of new drugs, which may eventually affect drug evaluation and approval processes. The resulting implications for the healthcare system and its stakeholders remain uncertain and require further investigation. Moreover, it is important to assess the readiness of all stakeholders to adapt to imminent changes in the future of healthcare. These are all considerations that warrant further exploration.

In 2020, the world gained a glimpse of RMPs’ potential with the rapid design and development of two COVID-19 mRNA vaccines. As noted above, RMPs have captured significant attention due to their ability to serve as a platform for the creation of effective treatments for a broad range of diseases beyond vaccines, filling gaps where gene and cell therapies have struggled. Furthermore, the simplicity and agility of RNA platforms could revolutionize the pharmaceutical industry across all stages of drug development, from bench to commercialization. To better illustrate this point, we will discuss how the unique attributes of the RNA platform may drive radical changes in operational approaches for pharmaceutical companies during drug development.

#### Drug design—discovering new treatment options

Historically, drug development has proceeded on a product-by-product basis that results in long, complex, and increasingly costly assessments that must be highly customized to the investigational product and disease. Consequently, the standard view in the industry has been that development programs proceed independently of one another.

The modularity of RNA platforms aims to change this paradigm by providing opportunities to streamline the drug development journey and exploit its shared processes. From a process perspective, RNA platforms will involve selecting the sequence, RNA construct, delivery vehicle, and route of administration most appropriate to the target of interest. The design and validation of each of these selections will inform future studies for new platform development initiatives.

The “plug-and-play” approach theoretically benefits RNA platforms. However, there are many potential challenges to overcome with current approaches, such as the need to enhance the protein yield to achieve clinical efficacy (especially for mRNA therapeutics and vaccines), which must also be considered in conjunction with the delivery system’s efficiency to protect and allow cellular uptake of mRNA^98^. Moreover, in vivo delivery systems still need to advance further to achieve specificity in tissue targeting^98^. For mRNA therapeutics and vaccines, chronic dosing will be another challenge as robust protein production is difficult to maintain over time with the production of antibodies against the protein or delivery system^98^. Novel mRNA platforms, such as saRNA and circRNA, have been developed as a solution to many of these challenges, and the field of mRNA therapeutics and vaccines is already seeing a rapid diversification in platforms beyond linear mRNA.

RNA therapies, due to their versatility and programmability, have the potential to modernize therapeutic approaches for rare and ultra-rare diseases affecting between 263 and 466 million people worldwide^99^. The development of ASOs and siRNAs exemplifies this potential, with these RNA molecules specifically designed to target genetic anomalies inherent in each disease^18,100^. ASOs, for instance, have been used to induce the degradation of transthyretin mRNA in the liver, reducing the production of abnormal proteins in hereditary transthyretin amyloidosis^101^. Similarly, siRNA has been utilized to interfere with the expression of the transthyretin protein, preventing its accumulation^38^. The concept of nano-rare applications and *n* = 1 ASOs also represents a significant advance in the field of RNA therapies. This approach involves the development of highly personalized therapies designed to treat individual patients with unique genetic profiles, as demonstrated in a case where a custom ASO drug, milasen, was developed for a single patient suffering from Batten disease^102^.

Looking to the future, information and big data will be essential components in RNA drug design and medical innovation. Given their inherent characteristics, RNA-based drugs are especially well suited to incorporate computational approaches and systematic learning processes^63,64^. As such, we may see an increased focus on artificial intelligence and genomic research in the development of RMPs. These computationally informed learnings can be used to design RNAs themselves or to identify encoded targets. For example, the ability to rapidly change the RNA sequence, along with advances in genomic sequencing and computation, allow for the rapid design of custom therapeutic solutions that can be extended to bring personalized cancer vaccines closer to becoming a reality. In this scenario, a patient with cancer can have their genome and transcriptome profiled using next-generation sequencing technologies and their data analyzed with machine learning to generate a custom RMP^103^. The versatility of RNA platforms, in conjunction with these powerful computational tools, also allows rapid alteration of the antigen sequence, which is essential in cases of rapid biological evolution, such as those found in viruses like SARS-CoV-2 and influenza. This flexibility allows for rapid adjustments to be made without having to go through the antiquated process of recombinant protein engineering, which can take weeks or months.

As computational tools evolve, RNA platforms will become more robust. In addition, machine learning can also be leveraged in epidemiology to predict and prepare for future pandemics and seasonal infections. A recent scenario from the COVID-19 pandemic highlights opportunities for advancement in this field: the rapid response to the Omicron variant, which was enabled by combining RNA’s flexibility with sequencing and epidemiology^104^. The accumulation of data and progression of analytical tools will open new horizons to advance existing technology and engineer novel therapeutics.

#### Clinical trials and regulation

Changes in clinical trials and regulatory processes during the COVID-19 pandemic may be a preview of how the industry could soon be reshaped. The pandemic reinforced the importance of broader adoption of master protocols for well-designed randomized clinical trials, alleviation of redundancies, and decentralized trial designs, increasing efficiencies while maintaining scientific rigor; some of these elements may become mainstays in the clinical landscape for platform technology.

Once the therapeutic versatility of mRNA is validated in additional clinical trials, this multiproduct platform will have the potential to streamline clinical assessments and enable faster approvals. To achieve this, a quality-by-design (QbD) approach could be implemented, which emphasizes building quality into the production process rather than increasing testing^105^. This approach is based on defining critical quality attributes (CQAs), allowing manufacturers to demonstrate consistency in both process and product for regulatory bodies. A production framework with QbD implementation would allow process design, manufacturing, and control strategy to be developed and approved as a standardized process^105^. This “prequalification step” would ultimately limit validation to a few studies and comparability checks for each product developed within a flexible platform, reducing approval timelines and enabling rapid response to emerging threats^105^.

Clinical trials may be streamlined even further once CQAs and QbD are standardized for greater utilization across geographies via master protocols and convergence of regulatory pathways. A master protocol can evaluate multiple investigational questions instead of via several separate clinical trials, thus accelerating drug development and increasing efficiency. Within this framework, a research ecosystem is created for a trial network where common standard operating procedures can be employed to generate data efficiently^106^. A master protocol will play an important role for trials evaluating platform technology, where standardized production processes are used to develop multiple therapeutics^106^. Moreover, these improvements will build on efficiencies already gained from innovative platform designs and the QbD approach discussed earlier. Some of the tactics used during the COVID-19 pandemic are also available as practical solutions to improve clinical trial efficiencies, such as telemedicine, remote monitoring and audits, local testing, consolidated regulatory evaluations and trial activation processes, and collaborative data sharing^107^.

Based on rapidity of clinical trial progressions for COVID-19 vaccines, it is anticipated that clinical trials evaluating platform biotechnology will continue to demonstrate accelerated progression due to the interchangeable nature of the platform. Nevertheless, it is crucial to establish safety and regulatory protocols that ensure quality, safety, and efficacy are evaluated appropriately^108^. It will be critical to control the purity, quality, and consistency of materials used in the production process^108^. Moreover, the purity of RNA and proportions of transcripts present, as well as the proportion of mRNA capped and polyadenylated, will be important to monitor^108^. Finally, quantification of encapsulated mRNA and size distribution of the particles should be monitored for manufacturing consistency, along with the usual parameters such as stability, identity, and sterility^108^. As with any therapeutic, systemic and local toxicity and inflammatory response will also need to be monitored with mRNA products in animal studies and clinical trials^108^.

The regulatory landscape for RNA-based drugs is actively shaped by regulatory bodies such as the FDA and the European Medicines Agency (EMA). Currently, RMPs are typically encompassed by guidance for categories such as vaccines (e.g., mRNA vaccines) and gene/cell therapies (gRNA/CRISPR)^109^. Guidance is also being developed for certain RNA classes under certain circumstances. The FDA, for example, has even developed specific guidance for ASOs, “Nonclinical Testing of Individualized Antisense Oligonucleotide Drug Products for Severely Debilitating or Life-Threatening Diseases,” which offers an accelerated nonclinical development pathway for ASOs designed for rare diseases that use well-known backbones (e.g., nusinersen) with established safety profiles^110,111^. However, those RMPs without clear guidance (e.g., RNAi) and those ASOs that do not use the nusinersen backbone often encounter regulatory challenges due to the lack of clear guidance. This absence can lead to uncertainties in development, safety concerns, and regulatory hurdles^109^.

#### Manufacturing—the “process is the product” is no longer

The expansion of the RNA drug space has catalyzed the need for increased manufacturing of mRNA to meet the growing global demand. However, the issue of limited manufacturing capacity is rapidly becoming a problem of the past. Thanks to the involvement of privately owned supply chains and CDMOs, the mRNA industry now has substantial manufacturing capacity that can deliver over 5 billion doses globally, enough to dose most of the world’s population. Furthermore, strategies are in place to establish local production of mRNA vaccines in specific geographic locations, such as Africa, to respond to emerging threats. Emerging technologies such as Touchlight’s doggybone DNA (dbDNA™) and automated RNA production may enable faster, more efficient mRNA manufacturing processes^112–114^. This points to a vast manufacturing potential in the market that can be leveraged to support the growth of the RMP industry.

The broad impact of RMPs in healthcare also stems from simpler and more agile manufacturing. Traditionally, vaccines have been considered to be complex biologics, where a rigid and difficult-to-replicate cell- or egg-based process defines the product. However, with RNA pharmaceuticals, we are entering an era where complex manufacturing processes no longer serve as barriers to entry that protect market shares beyond patent lifetime. While the RNA-based therapeutic production process is still complex for mRNA/LNP, its distinct advantage compared to the production of complex biomolecules is that the same processes can be repeated over and over for different products with minimal retooling.

RNA platforms are unlike those that have come before as RNA encoding novel proteins can be quickly made at clinical scale and quality with few small modifications in automated, modular manufacturing units. Once the molecular chemistry and delivery system for the product is established, drug design and synthesis can be accelerated^1,15^. RNA technology is also flexible; mRNA production does not require dedicated production lines for each therapeutic because mRNA platforms rely on the same base materials and use cell-free enzymatic processes^1,15^. This enables manufacturers to quickly adapt to newly identified pathogens, offering unparalleled levels of production flexibility. However, standardization of production and control methods remains a challenge, with many companies keeping their methods proprietary^108^. This allows manufacturing to readily adapt to newly identified pathogens. The modular nature of the mRNA therapeutic and vaccine platform offers unparalleled levels of production flexibility while streamlining production will remove many barriers to entry for the pharmaceutical industry. This will drastically accelerate the development of new products in the coming years, and “process is the product” will no longer apply as the ease of manufacturing RNA provides opportunities to revolutionize the market.

#### Commercial models

The flexibility of the RNA platform could enable a wide range of novel commercial possibilities. To illustrate this, we will explore the potential disruptions that may be made possible by manufacturers pursuing limited delivery vectors for ‘platform advancement’ strategies. In contrast to product-/indication-level tailoring of delivery vector components, such as lipids or polymers, manufacturers can instead use formulations that are close enough to existing products that, by FDA standards, previously collected data can be used in clinical/regulatory submissions. Such strategies have the potential to allow for accelerated development by enabling evidence generation at a platform level and creating formulation-/indication-level data packages for the underlying delivery platform. Importantly, there is already an industry precedent in RNA with Alynlam and their RNAi platform. By developing a repertoire of delivery vectors and referencing the data packets in each subsequent program, Alynlam was able to increase R&D capital efficiency and clinical success rate, resulting in a Phase 1 to launch success rate of 62.0%, much higher than the industry standard of 10.3%. Platform advancement strategies can be applied to vaccines as well. With the broader application of digital vaccines^115^, the drug development process can be condensed to the selection of the RNA sequence for a target disease and an already well-defined delivery vector formulation, therefore allowing for immediate entry into late-stage development with a focus solely on the assessment of the encoded target.

Platform advancement strategies have the potential to significantly change the nature of drug development; however, there are some hurdles to implementation that must be considered. As discussed above, regulators have yet to establish clear guidelines for most RMPs. Though this lack of guidance may appear to be an oversight, this methodical approach is likely due to the substantial repercussions that exist downstream. For example, rapid drug development will likely be hindered by the time required for each market to incorporate new products, resulting in implementation-related concerns that may lead to poor uptake at launch or overall market disruption. This is particularly true for mRNA-based vaccines, the primary focus of RNA-based products, which will be launched in the adult vaccine market that has been undergoing a renaissance many years in the making. The entrance of RNA-based platforms is expected to accelerate this revolution by reducing manufacturing complexities traditionally associated with vaccine development. This can be seen with Pfizer and Moderna’s influenza programs that have both advanced to late-stage development^116,117^, as well as Moderna’s respiratory syncytial virus (RSV) program that was submitted for regulatory approval in 2023^118^. However, the underlying infrastructure of the adult vaccine market may not be able to readily accommodate an influx of newly approved products in a future where innovation is concentrated in the adult vaccine segment. To keep up with these accelerated approvals, a more integrated and collaborative approach between industry, regulatory and public health bodies, and government agencies is essential for a holistic approach to drug development and approval processes.

Despite these potential hurdles, delivery vector platform advancement strategies offer many promising commercial benefits. Developing an ecosystem of products that share the same delivery vector platform and a coordinated promotional strategy could transform the focus of product promotion away from product- and indication-specific strategies and towards platform-specific ones. This has the potential to streamline communications with physicians, since established platforms will have already addressed safety concerns. It will also likely increase patient loyalty and brand recognition. Just as consumers have become accustomed to different smartphone ecosystems, patients may come to expect a similar experience with drug products as they trust certain delivery vector platforms and the manufacturers that provide them.

The shift to platform-based marketing and streamlined development timelines could have a significant impact on the healthcare system. First, it may become more efficient as patients receive targeted therapy for a specific disease rather than one-size-fits-all treatments. Additionally, by reducing production times and costs, RNA-based products may become more widely available and affordable for patients. This could result in improved health outcomes, particularly for diseases that are difficult to treat with traditional therapeutics. Furthermore, the platform-based approach could foster competition among drug developers, resulting in reduced costs for both manufacturers and patients.

#### Potential challenges

**Fig. 3:**
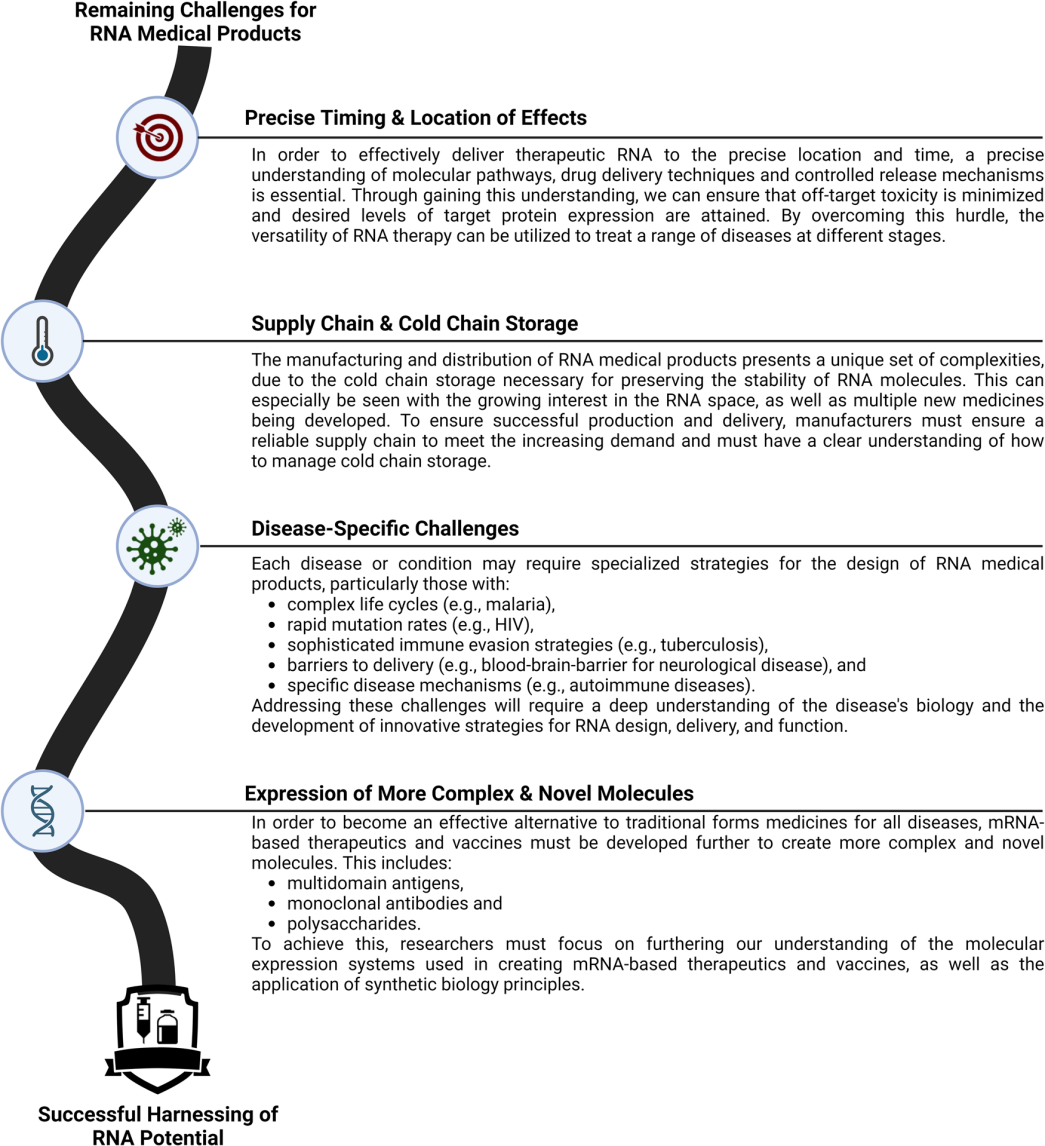
Navigating key challenges for the development of RNA medical products. Overview of the essential engineering challenges faced in the advancement of RNA-based medical products. These include achieving accurate timing and placement of therapeutic effects, overcoming supply chain and refrigeration issues, handling disease-specific difficulties, and developing the capacity to formulate intricate biomolecules like bacterial polysaccharides. “Created with BioRender.com”.

The potential for RNA-based medical products to revolutionize the healthcare landscape is undeniable. However, there are still numerous manufacturing challenges that need to be overcome before RMPs can reach their full potential (Fig. 3).

The path to COVID-19 vaccinations was a multidecade endeavor; the result was an unprecedented response to develop, approve, and administer a novel pharmaceutical agent in less than 2 years. Going forward, RNA products will face more traditional regulatory approval processes and manufacturing constraints without a global pandemic as a driving force. As such, pharmaceutical manufacturers developing RNA-based drugs will need to differentiate themselves from other market competitors, establish their superiority against a standard of care, or sufficiently demonstrate efficacy when a correlation has not been established. Manufacturers will also need to navigate the rapidly changing RMP landscape while building a platform with modular capabilities that assumes limited changes in the future. Supply chain and cold chain storage will also pose manufacturing challenges during clinical trials and post-regulatory approval. While the supply chain was strengthened to meet the demand for COVID-19 vaccine manufacturing, the impact of the launch of additional RNA pharmaceuticals and vast interest in the RNA space is uncertain. This will be further challenged by the cold chain storage associated with these pharmaceuticals, given the relative stability of RNA.

The main challenge in RMPs is to achieve precise timing and location of therapeutic effects rather than just short-term and limited impact. A key aspect of this challenge is to minimize off-target toxicity and unwanted immunogenicity while achieving the desired levels of target protein expression. Off-target effects of RNA therapeutics and vaccines can lead to unintended biological responses, which have been reviewed extensively^2,119,120^, thereby undermining the precision that makes RNA therapies attractive. Furthermore, recent studies have shown that mRNA vaccines lead to the production of IFN-I and proinflammatory cytokines that^121,122^, while stimulating immune responses and improving vaccine efficacy, may also result in immunological adverse effects^122^. Such unwanted responses could lead to side effects for patients, such as those observed following the administration of mRNA COVID-19 vaccines. Many recipients experienced some type of side effect, ranging from mild to severe^123^. While the localized reactions, such as swelling and redness at the injection site, are generally manageable, systemic responses can be more concerning. Rare, but serious adverse events, including myocardial infarction, stroke, or anaphylaxis, have also been reported in relation to these vaccines, highlighting the need for further investigation and monitoring of potential side effects^124^. To expand the number of treatable diseases using RNA’s versatility at different disease stages, we need to overcome this rate-limiting step. This requires a thorough comprehension of molecular mechanisms, drug delivery techniques, and controlled release.

In addition to these hurdles, another important consideration is the unique challenges that each disease or condition represents. For instance, significant implementation of synthetic biology will likely be needed before RNA-based approaches represent a viable competitor to glycoconjugate vaccines. While RNA provides a powerful platform for exploring and developing potential solutions, it should not be expected to revolutionize the pharmaceutical industry single-handedly. Rather, RNA provides a platform to explore and develop solutions, and leveraging information and technology will be essential in our approach to successfully harness RNA’s potential.

Compatibility with the immune system poses yet another challenge. Inefficient B-cell targeting, for example, impedes the development of broadly neutralizing vaccines for diseases such as SARS-CoV-2^125^, HIV^126^, and influenza^127^. Additionally, integrating robust humoral responses with effective T-cell-mediated immunity remains elusive, particularly at mucosal sites^128^. It is also vital not to overlook potential adverse effects linked to specific immune subsets, such as NK/monocyte subsets, dendritic cell subsets, and NK T-like cells. These subsets play dual roles, both enhancing the vaccine’s efficacy and contributing to side effects, underscoring the importance of achieving a balanced immune response for vaccine safety^122,129^. RMP components, such as LNPs, can also dictate immune system interactions. LNPs employed in mRNA vaccines, for example, possess distinctive physical and chemical properties that dictate their interactions with the immune system. Though LNPs can function as adjuvants to amplify specific immune responses, their potent immunogenicity can be a double-edged sword, potentially inciting inflammation^122^. Notably, LNPs, particularly those with ionizable lipids, have been found to stimulate proinflammatory cytokines and activate both antibody and T-cell responses^130–132^.

The experiences of BioMarin and Ionis Pharmaceuticals serve as case studies for such challenges. BioMarin’s RNA-based product Kyndrisa, aimed at treating Duchenne muscular dystrophy, fell short of FDA approval due to insufficient evidence of effectiveness, safety concerns, and a lack of dystrophin-positive fibers^133,134^. Similarly, despite Ionis Pharmaceuticals’ ION449 showing a significant reduction in LDL-C levels in Phase 2b SOLANO study, the drug did not meet the pre-specified efficacy criteria, resulting in the decision to cease its advancement to Phase 3^135^.

As with any new technology or approach, there are potential drawbacks and concerns. The rapid pace of development and approval may lead to regulatory oversights or gaps in data, which could impact patient safety. Additionally, the focus on platform-based marketing may lead to less attention and investment in rare diseases or niche markets that may not fit within a platform strategy. The emergence of platform-based marketing may also further exacerbate existing healthcare disparities, particularly if patients in underserved communities do not have access to the latest technologies or have limited options for treatment.

### Outlook

Advancements in mRNA have undoubtedly been a game-changer for COVID-19 vaccines. However, learnings from the pandemic will have far-reaching implications for future programs and therapeutic areas. There are strong indicators that RNA will continue to revolutionize the field, and we have yet to reach the “new normal” for the global pharmaceutical ecosystem. Interest in the sector has risen considerably, with investment dollars seeding new ventures and partnering dollars securing pharma access to innovative concepts, technologies, and assets. The expectations and efforts go well beyond mRNA vaccines, and RNA holds enormous promise in accelerating drug development across therapeutic areas. In our bold vision for the future of RNA, the industry will continue to adapt, RNA will continue to disrupt and challenge the status quo, and practical challenges will be overcome. RNA will become a critical pillar in the future of medicine.

### Supplementary information

Supplementary Information

## Data Availability

Financial data for public companies that support the meta-analysis of the current RMP landscape is available at Yahoo Finance with the identifier https://finance.yahoo.com/. Search terms for this analysis include names for companies specializing in RNA technology in the spaces of mRNA, microRNA, CRISPR, ASO, and siRNA (excluding diversified companies, such as Merck, Pfizer, and Sanofi). Stock prices were pulled on May 17, 2023, representing the market close price of each stock on the date of 12/31 from 2018 to 2022. Data for the value of RNA therapeutic and vaccine markets was obtained from Evaluate with the identifier https://www.evaluate.com/. Report filters for RNA therapeutics and RNA vaccines include generic name (patisiran, nusinersen, lumasiran, inclisiran, elasomeran, inotersen sodium, golodirsen, eteplirsen, volanesorsen, viltolarsen, casimersen, and vutrisiran) and Annual Sales WW (Exclude blank or zero rows, Top 20 ranked on field value (2028) Descending), or product name (Givlaari and Comirnaty). This data was collected as part of a report created for Pfizer, which is unavailable to the public. Researchers interested in accessing similar data may contact Evaluate Pharma directly at info@evaluate.com or explore institutional licensing options. Data for the number of programs by RNA class and mRNA pipeline by therapeutic area was obtained from Beacon Intelligence, with the identifier https://beacon-intelligence.com/. Data was obtained from the RNA dataset with the data view ‘Drug Data.’ This dataset is unavailable to the public. Researchers interested in accessing this or similar data may contact Beacon Intelligence directly at beacon@hansonwade.com or explore institutional licensing options. Data pertaining to RNA clinical programs is available at ClinicalTrials.gov with the identifier https://clinicaltrials.gov/. All other data supporting the meta-analysis was collected from Cortellis Drug Discovery Intelligence, a Clarivate product. The identifier is https://clarivate.com/. Product categories included in the query were: Product Categories included in the query were: mRNA Vaccines, Oligoribonucleotide (RNA), Small Nuclear RNA (snRNA), RNA Vaccines, RNA Interference, Aptamers, mRNA, Single Guide RNA (sgRNA), Small Interfering RNA (SiRNAs), Small Activating RNA (saRNA), Self-Amplifying mRNA Vaccines, Self-Amplifying RNA Vaccines, Short Hairpin RNA (shRNA), Single-stranded oligoribonucleotide (RNA), Small Nuclear RNA (snRNA) U1, Small Nuclear RNA (snRNA) U7, MicroRNA, mRNA-Based Gene Therapy, Long Non-Coding RNA (lncRNA), Double-Stranded RNA (dsRNA), Double-stranded oligoribonucleotide (RNA), and DNA-Directed RNA Interference (ddRNAi). This dataset is unavailable to the public. Researchers interested in accessing this or similar data may contact Clarivate directly or explore institutional licensing options.
