# Supplementary material for: Breaking the mold with RNA—a “RNAissance” of life science
Source: NPJ Genom Med. 2024 Jan 9;9:2. doi: 10.1038/s41525-023-00387-4 (PMC10776758; doi:10.1038/s41525-023-00387-4)
Supplement: Supplementary file 1 — Supplementary Information [file 41525_2023_387_MOESM1_ESM.pdf]

Supplementary Information

Supplementary Box 1. Milestone Advances in mRNA Influenza Vaccines

The past decade has seen considerable progress in the research and development of mRNA influenza vaccines. Moderna made early strides by initiating the first clinical trials for two monovalent mRNA vaccines. H10N8 and H7N9, respectively.[1-3] The trials, which were completed in 2018, provided a proof-of-concept for the safety and immunogenicity mRNA influenza vaccines in healthy adults. Not long after, in 2018, Pfizer and BioNTech initiated their joint venture into influenza mRNA vaccine development.[4] However, development on these influenza vaccines was put on hold by the COVID-19 pandemic as resources and attention were shifted to create the mRNA COVID-19 vaccines.

The success of mRNA COVID-19 vaccines, including those developed by Moderna and Pfizer/BioNTech, demonstrated the potential of mRNA-based platforms. Combined, the companies have delivered nearly 1 billion doses (including boosters) in the United States alone as of May 11th, 2023.[5,6] This achievement led to the revival of interest in mRNA influenza vaccines. Both Moderna and Pfizer/BioNTech incorporated their successful COVID-19 mRNA platforms into the design of their influenza vaccines and initiated trials in 2021.[7,8]

Moderna's quadrivalent flu vaccine, mRNA-1010, showed promising results by increasing antibody titers against all four targeted virus strains in healthy adults.[9] However, the vaccine's protective efficacy varied among the strains, which suggests that mRNA vaccines containing only HA may not be superior to currently available influenza vaccines.[10] Pfizer/BioNTech recently released early clinical data on their quadrivalent modRNA vaccine, qIRV (22/23), which demonstrated that the qIRV (22/23) vaccine can stimulate robust T cell responses. These responses could potentially provide enhanced protection against severe disease. Both the Moderna and Pfizer/BioNTech’s quadrivalent vaccines have now entered Phase 3 trials.[11,12]

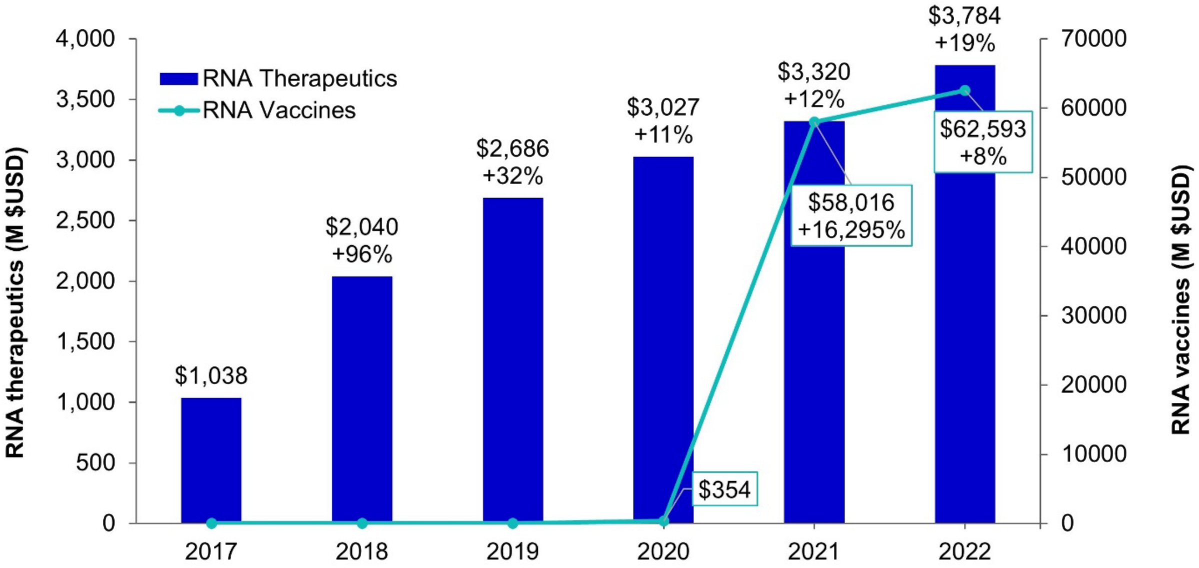

Supplementary Figure 1: Annual revenues of currently available RNA-based vaccines and therapeutics

Annual revenues of RNA therapeutics contrasted with the annual revenues of RNA vaccines. Percent growth for both RNA therapeutics and RNA vaccines are presented from each previous year. This includes data for Patisiran, Givosiran, Nusinersen, Lumasiran, Inclisiran, Elomeran, Tozinameran, Inotersen, Golodirsen, Eteplirsen, Volanesorsen, Viltolarsen, Casimersen and excludes data for Mipomersen, Pegaptanib, Defitelio, Fomivirsen and Rintatolimod. Data was obtained from Evaluate Pharma, accessed April 1st, 2023.

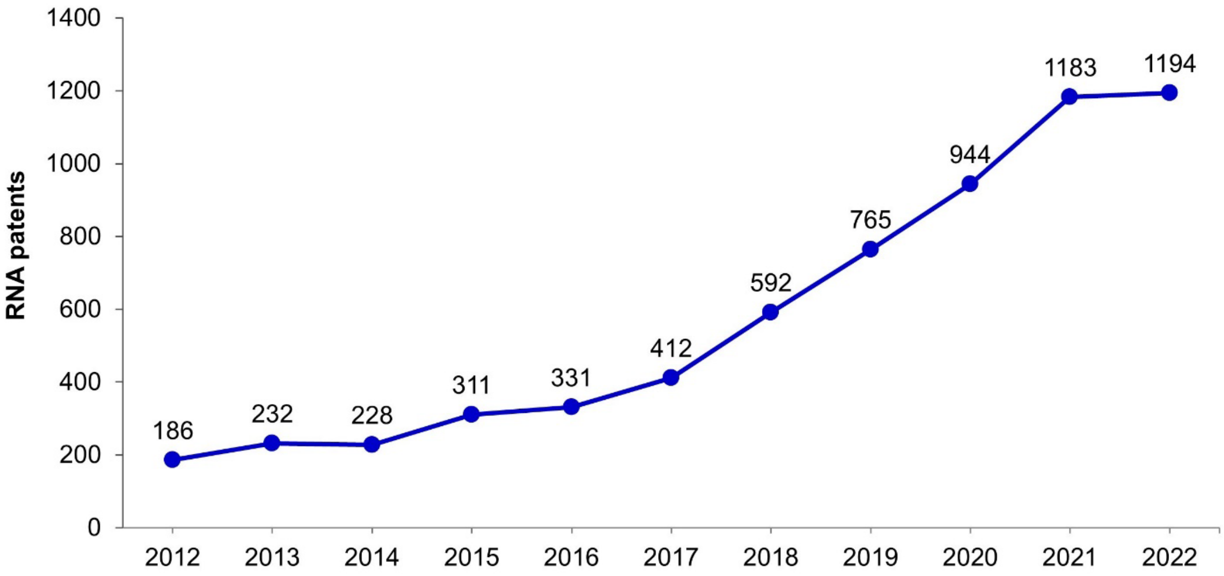

Supplementary Figure 2: RNA-related IP activity over time

Number of RNA-related patents filed by year. Data was obtained from Cortellis Drug Discovery Intelligence, accessed April 1st, 2023. Product Categories included in the query were: mRNA Vaccines, Oligoribonucleotide (RNA), Small Nuclear RNA (snRNA), RNA Vaccines, RNA Interference, Aptamers, mRNA, Single Guide RNA (sgRNA), Small Interfering RNA (SiRNAs), Small Activating RNA (saRNA), Self-Amplifying mRNA Vaccines, Self-Amplifying RNA Vaccines, Short Hairpin RNA (shRNA), Single stranded oligoribonucleotide (RNA), Small Nuclear RNA (snRNA) U1, Small Nuclear RNA (snRNA) U7, MicroRNA, mRNA-Based Gene Therapy, Long Non-Coding RNA (lncRNA), Double Stranded RNA (dsRNA), Double stranded oligoribonucleotide (RNA), and DNA-Directed RNA Interference (ddRNAi).

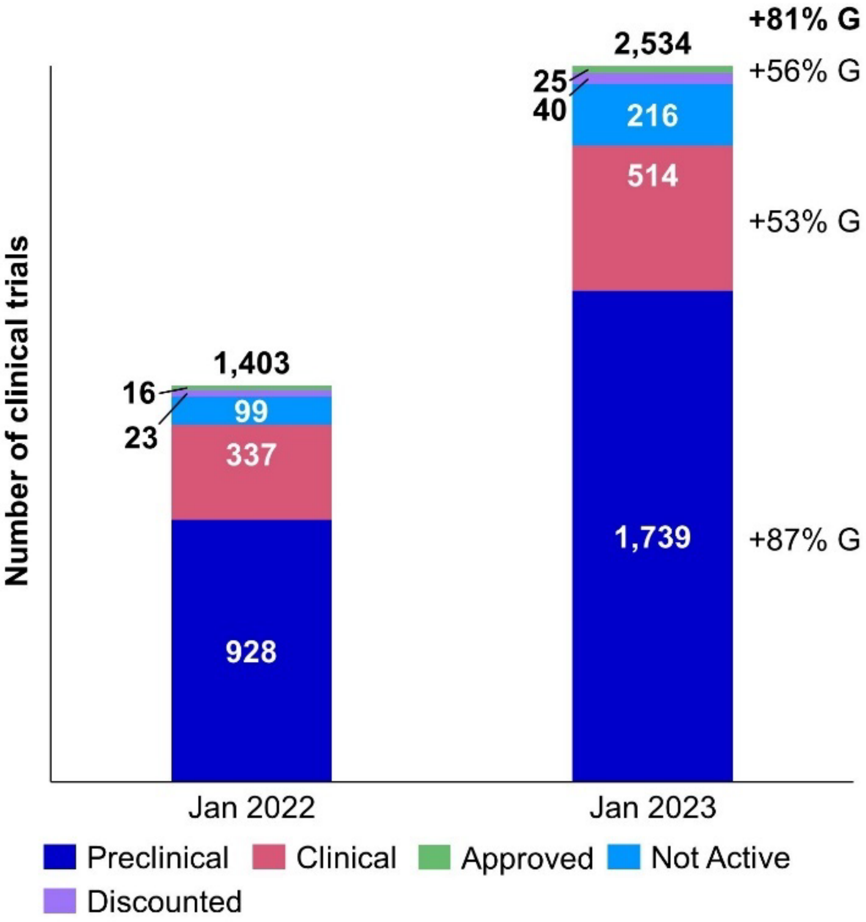

Supplementary Figure 3: Number of RNA-related programs by development status

Number or RNA-related programs, broken down by development status. Percent growth is provided for each development status category from January 2022 to January 2023. Data was obtained from Beacon Intelligence, RNA dataset, accessed April 1st, 2023. Data View used in the RNA dataset was ‘Drug Data.’ Standard statistical methods were used in the analysis of the data.

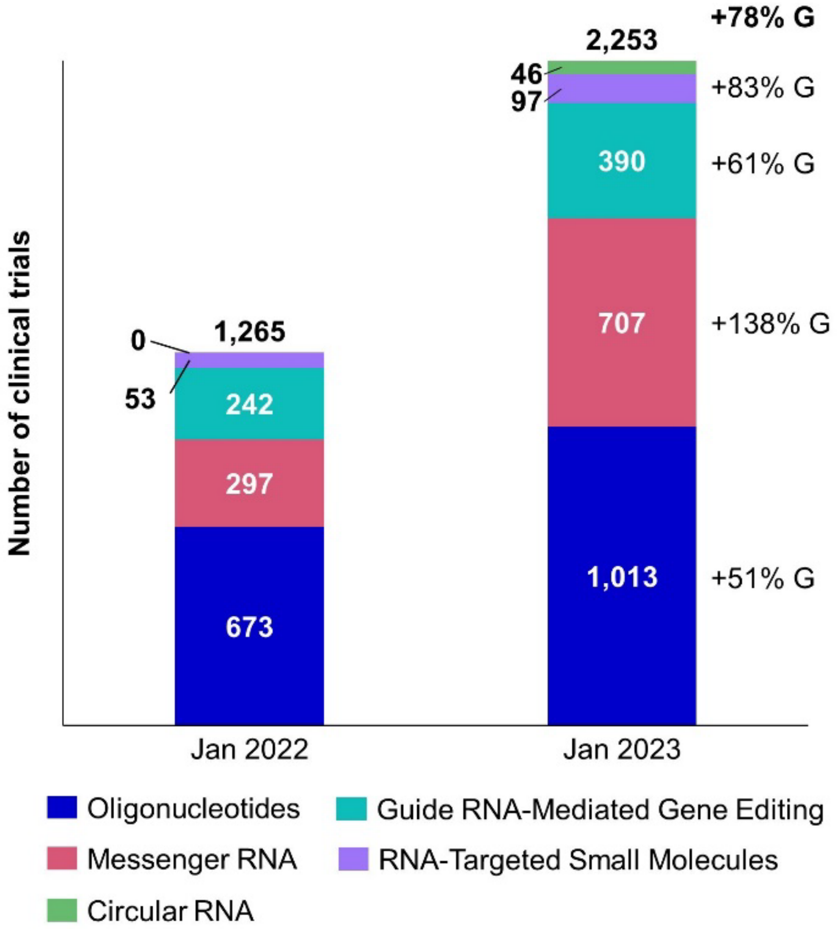

Supplementary Figure 4: Number of clinical programs by RNA class

Number or RNA-related clinical programs, broken down by RNA class. Percent growth is provided for each category from January 2022 to January 2023. Data was obtained from Beacon Intelligence, RNA dataset, accessed April 1st, 2023. Data View used in the RNA dataset was 'Drug Data.' Standard statistical methods were used in the analysis of the data.

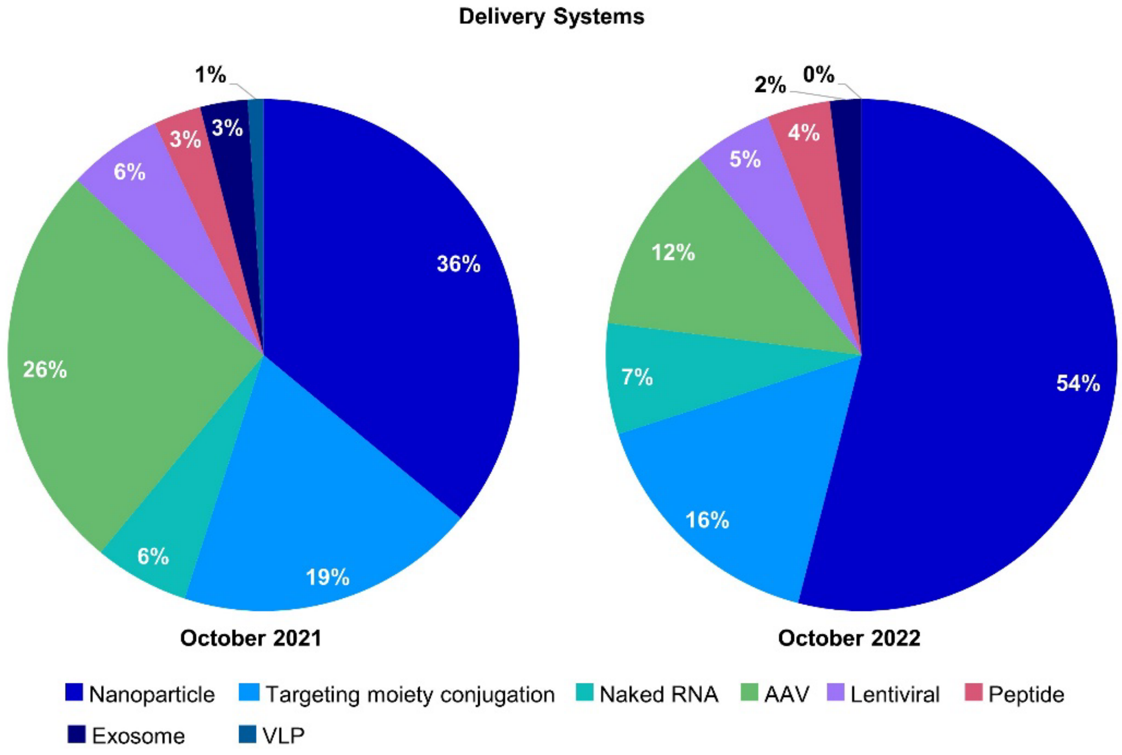

Supplementary Figure 5: Number of clinical programs by delivery systems

Number or RNA-related clinical programs, broken down by RNA delivery system, from October 2021 to October 2022. Abbreviations: AAV, adeno-associated viruses; VLP, viral-like particle. Data was obtained from Beacon Intelligence, RNA dataset, accessed October 12, 2022. Data View used in the RNA dataset was 'Drug Data.'

Supplementary Table 1: List of promising RNA therapeutics and vaccines in clinical trials

Drug	Disease/Target	MOA	ROA	Status	Last update	Clinical Trial Number
**ASO**						
CDK-004	Hepatocellular Carcinoma	Reduce inhibition of cancer cell recognition	IV	Phase I	Mar 2023	NCT05375604
NIO752	PSP	Inhibition of Tau translation	IT	Phase I	Apr 2023	NCT04539041 NCT05469360
RO7248824	Angelman Syndrome	Reduce ubiquitin-protein ligase E3A silencing	IT	Phase I	Apr 2023	NCT04428281
QRL-201	ALS	Restore STMN2 function	IT	Phase I	Apr 2023	NCT05633459
QRL-101	ALS	Restore Kv7 function	PO	Phase I	Apr 2023	NCT05667779
WVE-N531	DMD	Exon-skipping	IV	Phase I/II	Jul 2022	NCT04906460
WVE-003	Huntington Disease	Reduce HD-specific mutant huntingtin	IT	Phase I/II	Jan 2023	NCT05032196
SQY51	DMD	Exon-skipping	IV	Phase I/II	Mar 2023	NCT05753462
Eplontersen	Amyloid Cardiomyopathy	Reduce TTR protein expression	SC	Phase III	Mar 2023	NCT05667493
**siRNA**						
ARO-HIF2	ccRCC	Deregulation of HIF2A	IV	Phase I	Jul 2022	NCT04169711
APN401	Cancer (multiple forms)	Inhibition of Cbl-b	IV	Phase I	Aug 2022	NCT03087591
STP705	Cutaneous BCC	TGF-β1 and COX-2 inhibition	Intrascar	Phase I/II	Mar 2023	NCT05196373
PSCT19	Hematological Malignancies	Down-regulation of PD-L1/PD-L2	IV	Phase I/II	Apr 2021	NCT02528682
siG12D LODER	Pancreatic Cancer	Inhibits KRAS expression	IV	Phase II	Jul 2021	NCT01676259
ARO-HBV	Hepatitis B	Inhibition of virus	SC	Phase II	Apr 2021	NCT03365947
ALN-HSD	NASH	HSD17B13 inhibition	SC	Phase II	Apr 2023	NCT05519475
BMT101	Hypertrophic Scar	Down-regulation of CTGF	ID	Phase II	Jul 2023	NCT04012099
SYL1801	Macular Degeneration	Down-regulation of NRARP	Topical	Phase II	Jan 2023	NCT05637255
MIR 19	COVID-19	Inhibition of viral RdRp	INH	Phase II/III	Mar 2023	NCT05783206
SYL1001	Sjögren Syndrome	Down-regulation of TRPV1	Topical	Phase III	Feb 2023	NCT04819269
Fitusiran	Hemophilia	Down-regulation of Thrombin	SC	Phase III	Apr 2023	NCT05662319
Lumasiran	PH1	Down-regulation of HAO1	SC	Phase III	Apr 2023	NCT04152200 NCT03905694
Inclisiran	FH	Down-regulation of PCSK9	SC	Phase III	Apr 2023	NCT05682378, NCT05118230
DCR-PHXC	PH	Down-regulation of LDHA	SC	Phase III	Jun 2022	NCT03365947
Cemdisiran	GMG, PNH	Down regulation of C5a Receptor	SC	Phase III	May 2023	NCT05070858 NCT05744921

Drug	Disease/Target	MOA	ROA	Status	Last update	Clinical Trial Number
**Aptamer**						
AON-D21	Undetermined	Inhibits complement component C5a	IV	Phase I	Dec 2022	NCT05343819
ApTOLL	Acute Ischemic Stroke	TLR4 antagonist	IV	Phase I/II	Sep 2023	NCT04734548
NOX-A12	Metastatic Pancreatic Cancer, Glioblastoma	Disrupts CXCR4-CXCL12 interactions	IV	Phase II	Feb 2023	NCT04901741
BT200	VWD, Hemophilia A	Blocks the clearance of VWF antigen	SC	Phase II	Nov 2021	NCT04677803
Zimura	GA, Macular Degeneration	Inhibits Complement C5	IVT	Phase III	Mar 2023	NCT04435366
**mRNA**						
mRNA-3745	GSD	G6Pase activity	IV	Phase I	Mar 2023	NCT05095727
mRNA-1189	EBV	Immune response to virus	IM	Phase I	Feb 2023	NCT05164094 NCT05831111
BNT112	Prostate Cancer	Immune response to cancer	IV	Phase I/II	Mar 2023	NCT04382898
MRT5005	Cystic Fibrosis	Restores CFTR expression	Nebulization	Phase I/II	Nov 2020	NCT03375047
mRNA-3927	Propionic Acidemia	mitochondrial enzyme PCC activity	IV	Phase I/II	Jan 2023	NCT05130437 NCT04159103
BNT111	Malignant melanoma	Immune response to cancer	IV	Phase II	Apr 2023	NCT04526899
AZD8601	Heart Failure	Restores VEGF-A expression	Epicardial Injection	Phase II	Aug 2021	NCT03370887
BNT113	Head and Neck Cancer	Immune response to cancer	IV	Phase II	May 2023	NCT04534205
BNT122	Colorectal Cancer	Immune response to cancer	IV	Phase II	May 2023	NCT04486378
mRNA-1345	Respiratory Syncytial Virus	Immune response to virus	IM	Phase II/III	Jan 2023	NCT05127434
mRNA-1647	Cytomegalovirus	Immune response to virus	IM	Phase III	Apr 2023	NCT05085366
mRNA-1010	Influenza	Immune response to virus	IM	Phase III	May 2023	NCT05827978
qIRV	Influenza	Immune response to influenza	IM	Phase III	Apr 2023	NCT05540522
**miRNA**						
MRG-110	Wound treatment	miR-155 inhibitor	ID	Phase I	May 2019	NCT03603431
RGLS4326	Polycystic Kidney Disease	miR-17 inhibitor	SC	Phase I	Dec 2021	NCT04536688
Remlarsen	Keloid	miR-29 mimic	ID	Phase I	Aug 2021	NCT03601052
RG-012	Alport Syndrome	miR-21 inhibitor	SC	Phase I	Apr 2022	NCT03373786
CDR132L	Myocardial Infarction, Acute Heart Failure	miR-132 inhibitor	IV	Phase II	Feb 2023	NCT05350969

Abbreviations: ALS = Amyotrophic lateral sclerosis; ASO = Antisense Oligonucleotide; BCC = Basal Cell Carcinoma; Cbl-b = Casitas B lymphoma-b; ccRCC = Clear Cell Renal Cell Carcinoma; CFTR = cystic fibrosis transmembrane conductance regulator COVID-19 = Coronavirus Disease 2019; COX-2 = cyclooxygenase-2; CTGF = connective tissue growth factor; DMD = Duchenne Muscular Dystrophy; EBV = Epstein-Barr Virus; FH = Familial Hypercholesterolemia; GA = Geographic Atrophy; GMG = Generalized Myasthenia

Gravis; GSD = Glycogen Storage Disease; HAO1 = Hydroxyacid Oxidase 1; ID = intradermal; IM = intramuscular; INH = inhalation; IT = intrathecal; IV = intravenous; IVT = intravitreal; KRAS = Kirsten rat sarcoma virus; MOA = Mechanism of action; NASH = Nonalcoholic Steatohepatitis; NRARP = NOTCH regulated ankyrin repeat protein; PCC = propionyl-CoA carboxylase; PCC = Propionyl-CoA carboxylase; PCSK9 = proprotein convertase subtilisin/kexin type 9; PD-L1 = Programmed death-ligand 1; PD-L2 = Programmed death-ligand 2; PH1 = Primary Hyperoxaluria Type 1; PNH = Paroxysmal Nocturnal Hemoglobinuria; PO = oral; PSP = Progressive Supranuclear Palsy; RdRp = RNA-dependent RNA polymerase; SC = subcutaneous; siRNA = Small interfering RNA; TLR=Toll-like receptor; TGF-β = Transforming Growth Factor-β; TRPV1 = Transient receptor potential vanilloid 1; TTR = Transthyretin; VEGF = Vascular endothelial growth factor; VWD = Von Willebrand Diseases; VWF = von Willebrand factor.

Note: Only products with activity in the past five years have been included.

Source: ClinicalTrials.gov (accurate up to May 16th, 2023)

Supplementary Information References:

1 Feldman, R. A. *et al.* mRNA vaccines against H10N8 and H7N9 influenza viruses of pandemic potential are immunogenic and well tolerated in healthy adults in phase 1 randomized clinical trials. *Vaccine* **37**, 3326-3334 (2019).

2 ClinicalTrials.gov. *Safety, Tolerability, and Immunogenicity of VAL-506440 in Healthy Adult Subjects (NCT03076385)*, https://www.clinicaltrials.gov/ct2/show/NCT03076385?term=H10N8&cond=Influenza&draw=2&rank=1 (2022)

3 ClinicalTrials.gov. *Safety, Tolerability, and Immunogenicity of VAL-339851 in Healthy Adult Subjects (NCT03345043)*, https://www.clinicaltrials.gov/ct2/show/NCT03345043?term=H7N9%3B+Moderna&cond=Influenza&draw=2&rank=1 (2021)

4 Pitts, J., Dunn, B. *Pfizer Starts Study of mRNA-Based Next Generation Flu Vaccine Program*, https://www.pfizer.com/news/press-release/press-release-detail/pfizer-starts-study-mrna-based-next-generation-flu-vaccine (2021).

5 CDC. *COVID Data Tracker*, https://covid.cdc.gov/covid-data-tracker/#vaccinations_vacc-total-delivered-total (2023).

6 Our World in Data. *COVID-19 vaccine doses administered by manufacturer*, https://ourworldindata.org/grapher/covid-vaccine-doses-by-manufacturer (2023).

7 ClinicalTrials.gov. *A Study to Evaluate the Safety, Tolerability, and Immunogenicity of a Modified RNA Vaccine Against Influenza (NCT05052697)*, https://www.clinicaltrials.gov/ct2/show/NCT05052697?term=Pfizer%3B+RNA&cond=Influenza&draw=2&rank=3 (2023)

8 ClinicalTrials.gov. *A Study of mRNA-1010 Seasonal Influenza Vaccine in Healthy Adults (NCT04956575)*, https://www.clinicaltrials.gov/ct2/show/NCT04956575?term=mRNA-1010&draw=2&rank=4 (2023)

9 Hussey, C., Talukdar, L. *Moderna Announces Positive Interim Phase 1 Data For mRNA Flu Vaccine and Provides Program Update*, https://investors.modernatx.com/news/news-details/2021/Moderna-Announces-Positive-Interim-Phase-1-Data-for-mRNA-Flu-Vaccine-and-Provides-Program-Update/default.aspx (2021).

10 Pecetta, S. & Rappuoli, R. mRNA, the beginning of a new influenza vaccine game. *PNAS* **119**, e2217533119 (2022).

11 ClinicalTrials.gov. *A Study to Evaluate a Modified RNA Vaccine Against Influenza in Adults 18 Years of Age or Older (NCT05540522)*,

https://www.clinicaltrials.gov/ct2/show/NCT05540522?term=Pfizer%3B+RNA&cond=Influenza&draw=2&rank=4 (2023)

12 ClinicalTrials.gov. *A Study of mRNA-1010 Seasonal Influenza Vaccine in Adults (NCT05415462)*, https://www.clinicaltrials.gov/ct2/show/NCT05415462?term=mRNA-1010&draw=2&rank=3 (2023)
